# Acute infection with *Brachyspira hyodysenteriae* affects mucin expression, glycosylation, and fecal MUC5AC

**DOI:** 10.3389/fcimb.2022.1042815

**Published:** 2023-01-06

**Authors:** Susanne Je-Han Lin, Emma T. Helm, Nicholas K. Gabler, Eric R. Burrough

**Affiliations:** ^1^ Department of Veterinary Pathology, Iowa State University, Ames, IA, United States; ^2^ Department of Animal Science, Iowa State University, Ames, IA, United States; ^3^ Department of Veterinary Diagnostic and Production Animal Medicine, Iowa State University, Ames, IA, United States

**Keywords:** *Brachyspira*, swine dysentery, fucose, sialic acid, GlcNAc, glycosylation, MUC2, MUC5AC

## Abstract

**Introduction:**

Infection with strongly *β-hemolytic* strains of *Brachyspira hyodysenteriae* leads to swine dysentery (SD), a production-limiting disease that causes mucohemorrhagic diarrhea and typhlocolitis in pigs. This pathogen has strong chemotactic activity toward mucin, and infected pigs often have a disorganized mucus layer and marked *de novo* expression of MUC5AC, which is not constitutively expressed in the colon. It has been shown that fucose is chemoattractant for *B. hyodysenteriae*, and a highly fermentable fiber diet can mitigate and delay the onset of SD.

**Methods:**

We used lectins targeting sialic acids in α-2,6 or α-2,3 linkages, N-acetylglucosamine (GlcNAc), α-linked L-fucose, and an immunohistochemical stain targeting N-glycolylneuraminic acid (NeuGc) to investigate the local expression of these mucin glycans in colonic tissues of pigs with acute SD. We used a commercial enzyme-linked immunosorbent assay (ELISA) to quantify fecal MUC5AC in infected pigs and assess its potential as a diagnostic monitoring tool and RNA *in situ* hybridization to detect IL-17A in the colonic mucosa.

**Results:**

Colonic mucin glycosylation during SD has an overall increase in fucose, a spatially different distribution of GlcNAc with more expression within the crypt lumens of the upper colonic mucosa, and decreased expression or a decreased trend of sialic acids in α-2,6 or α-2,3 linkages, and NeuGc compared to the controls. The degree of increased fucosylation was less in the colonic mucosa of pigs with SD and fed the highly fermentable fiber diet. There was a significant increase in MUC5AC in fecal and colonic samples of pigs with SD at the endpoint compared to the controls, but the predictive value for disease progression was limited.

**Discussion:**

Fucosylation and the impact of dietary fiber may play important roles in the pathogenesis of SD. The lack of predictive value for fecal MUC5AC quantification by ELISA is possibly due to the presence of other non-colonic sources of MUC5AC in the feces. The moderate correlation between IL-17A, neutrophils and MUC5AC confirms its immunoregulatory and mucin stimulatory role. Our study characterizes local alteration of mucin glycosylation in the colonic mucosa of pigs with SD after B. *hyodysenteriae* infection and may provide insight into host-pathogen interaction.

## 1 Introduction

Swine dysentery (SD) is a re-emerging and production-limiting disease in grower-finisher pigs caused by strongly β-hemolytic *Brachyspira* species such as *B. hyodysenteriae*, *B. hampsonii*, and *B. suanatina* (Burrough, 2017; Hampson and Burrough, 2019). Affected pigs develop mucohemorrhagic diarrhea and typhlocolitis, often with extensive fibrinous exudate, mucus, and blood in the large intestine, leading to reduced weight gain and considerable economic losses (Burrough, 2017; Hampson and Burrough, 2019). Infection by *B. hyodysenteriae* in pigs may regulate mucin composition in the colon, increase binding sites for the spirochetes, and alter the mucus layer with loss of striated organization (Quintana-Hayashi et al., 2015; Quintana-Hayashi et al., 2019). The mucus layer is mainly composed of mucins, which are large proteins that carry clusters of glycans with a high degree of structural complexity and form abundant recognition motifs (Moran et al., 2011; Li and Chai, 2019). Common structural elements of peripheral glycans include N-acetylgalactosamine (GalNAc), N-acetylglucosamine (GlcNAc), galactose, fucose, and sialic acids (Moran et al., 2011). Pigs inoculated with *B. hyodysenteriae* have shown increased sialomucin and decreased sulfomucin expression as assessed by histochemistry employing high iron diamine-alcian blue staining (Wilberts et al., 2014b; Lin et al., 2021). *In vitro* studies have also demonstrated that *B. hyodysenteriae* growth is increased in the presence of free sialic acid and GlcNAc, but not in the presence of other monosaccharides including galactose, GalNAc, and fucose (Quintana-Hayashi et al., 2019). Sialic acids are 9-carbon backbone α-keto acid sugars widely distributed in animal tissues and are often utilized by pathogens for binding to the host cells (Haines-Menges et al., 2015). The two most common sialic acids in mammals are N-acetylneuraminic acid (NeuAc) and its hydroxylated form N-glycolylneuraminic acid (NeuGc), and healthy pigs have an equal proportion of NeuGc and NeuAc (Venkatakrishnan et al., 2017). It has been proposed that sialic acids serve as an adhesion epitope for *B. hyodysenteriae* in its interaction with the colon, and its binding ability was found to be associated with a higher abundance of NeuGc-based structures on mucins instead of NeuAc (Venkatakrishnan et al., 2017; Quintana-Hayashi et al., 2019). While it has been shown that fucose could induce a chemotactic response from *B. hyodysenteriae* (Kennedy and Yancey, 1996; Quintana-Hayashi et al., 2021), overall mucin fucosylation was decreased in pigs inoculated with *B. hyodysenteriae* (Venkatakrishnan et al., 2017), and bacterial growth was not enhanced in the presence of fucose (Quintana-Hayashi et al., 2019). The role of fucose in the pathogenesis of SD remains unclear. Given that the previous findings were conducted with *in vitro* studies or using tandem mass spectrometry for mucin analysis, herein we use an immunohistochemical stain targeting NeuGc, and lectins derived from *Sambucus nigra* (SNA) targeting NeuAc attached to terminal galactose in an α-2,6 linkage, *Maackia Amurensis* II (MAL II) targeting NeuAc in an α-2,3 linkage, *Griffonia simplicifolia II* (GS-II) targeting GlcNAc, and *Lotus tetragonolobus* (LTL) targeting α-linked L-fucose containing oligosaccharides to investigate the local expression of these mucin glycans in colonic tissues of pigs with acute SD.

Pigs infected with *B. hyodysenteriae* commonly have excessive mucus production and remarkable mucin alterations in the large intestine (Wilberts et al., 2014b; Hampson and Burrough, 2019), which serve as a response to stimuli from the microbial products and inflammatory cytokines (Linden et al., 2008a). Mucins are a family of glycoproteins produced by mucin-producing cells, mainly goblet cells, which comprise the major constituent of mucus gel layers and serve as a defensive barrier on organs and tissues (Linden et al., 2008b; Corfield, 2015). Mucin 2 (MUC2) and mucin 5AC (MUC5AC) are both gel-forming mucins. While MUC2 is extensively found throughout the small and large intestine, MUC5AC is constitutively expressed in the respiratory tract, stomach, and gallbladder of pigs but not in the colon of healthy pigs (Kim et al., 2011; Quintana-Hayashi et al., 2015; Ushio et al., 2020). In pigs inoculated with *B. hyodysenteriae*, there is marked *de novo* expression of MUC5AC and increased MUC2 (Wilberts et al., 2014b; Quintana-Hayashi et al., 2015). Previously we have used RNA *in situ* hybridization (ISH) to demonstrate the expression of the pro-inflammatory cytokine interleukin (IL) 1-β in colonic tissues from pigs infected with *B. hyodysenteriae* since it has been shown to contribute to the expression of MUC2, MUC5AC, or both in humans (Kim et al., 2000; Kim et al., 2002; Song et al., 2003; Eastaff-Leung et al., 2010; Lin et al., 2021). Despite a tendency for increased expression in pigs with SD, we did not find statistical significance or association with MUC5AC or MUC2 expression and the expression of IL-1β. In human airway diseases, IL-17A has been found to play a critical role in regulating the expression of MUC5AC through the NF-κB and ERK1/2 pathway activation and act1-mediated signaling pathway (Chen et al., 2003; Fujisawa et al., 2009; Xia et al., 2014; Montalbano et al., 2016). IL-17A is a signature cytokine of type 17 T helper (Th17) cells, which is known to be crucial in immune surveillance and innate immunity at mucosal and barrier surfaces (Miossec and Kolls, 2012; Song et al., 2016). Besides lymphocytes, it is produced by natural killer T cells, innate lymphocytes, and occasionally neutrophils. *In vitro* studies have shown IL-17A had a stimulatory effect on mucin production in pigs infected with *B. hyodysenteriae*, and the upregulation of the *IL-17A* gene was associated with the MUC5AC protein abundance in the colonic tissue (Quintana-Hayashi et al., 2017). In the current study, we wanted to localize the expression of IL-17A in the colonic tissues of pigs inoculated with *B. hyodysenteriae* by using RNA ISH, and we expected to see an increased expression of IL-17A that may be associated with the *de novo* expression of MUC5AC in these pigs.

Definitive disease diagnosis of SD is typically based on selective anaerobic culture with isolation of a strongly β-hemolytic *Brachyspira* spp. that produces a positive ring phenomenon from the colonic mucosa or feces of clinically affected pigs, by polymerase chain reaction (PCR) assays, or a combination of both assays (Hampson and Burrough, 2019). Fluorescent ISH assays have also been developed and can aid the identification of *B. hyodysenteriae* and *B. hampsonii* in formalin-fixed tissues (Burrough et al., 2013; Wilberts et al., 2015; Hampson and Burrough, 2019). However, a diagnostic tool that would help predict or precede disease development, or aid in monitoring disease progression is lacking. One goal of this study was to use a commercial enzyme-linked immunosorbent assay (ELISA) to measure the amount of MUC5AC in the feces of pigs inoculated with *B. hyodysenteriae* as the disease progresses post-inoculation and to thereby evaluate its potential as a diagnostic monitoring tool.

Dietary fiber affects gut microbiome, mucin *O*-glycosylation, and intestinal barrier function (Desai et al., 2016; Ferrandis Vila et al., 2018; Gamage et al., 2020), and impacts the incidence and severity of disease expression after *B. hyodysenteriae* challenge (Pluske et al., 1996; Wilberts et al., 2014a). In general, a higher percentage of fermentable fiber may be beneficial to pigs for those diets that are based on less highly digestible ingredients (barley, triticale) (Hansen et al., 2010; Hansen et al., 2011), and pigs fed a poorly fermentable fiber diet with high insoluble fibers such as the biofuel coproducts like distillers dried grain with soluble (DDGS) present with a quicker onset of SD and higher disease incidence when challenged with strongly β-hemolytic *Brachyspira* species (Wilberts et al., 2014a). Conversely, a recent study suggested that expression of SD was likely not strongly associated with fiber type (Lee et al., 2022). The discrepancy in findings among these studies is likely due to the differences in experimental designs and the specific source of dietary fiber. Our previous studies have shown that replacing insoluble fiber with highly fermentable fibers delays the onset of SD and mitigates the presentation of SD (Helm et al., 2020; Helm et al., 2021). Herein, we used specimens from one of the animal inoculation experiments for further histopathology experiments.

The objectives of the present study were fourfold: 1) to quantify α-2,6 and α-2,3 linked NeuAc, NeuGc, GlcNAc, and α-linked L-fucose expression, and their correlations with MUC5AC and MUC2 expression, 2) to evaluate the expression of IL-17A in the colonic mucosa of pigs with acute SD relative to uninfected controls, 3) to measure the quantity of MUC5AC in feces of pigs with acute SD as the disease progresses and to evaluate its potential as a diagnostic monitoring tool, and 4) to determine if replacement of dietary insoluble fiber with highly fermentable fibers would change the presentation of the above parameters.

## 2 Materials and methods

### 2.1 Animal study and source of samples

**Figure 1:**
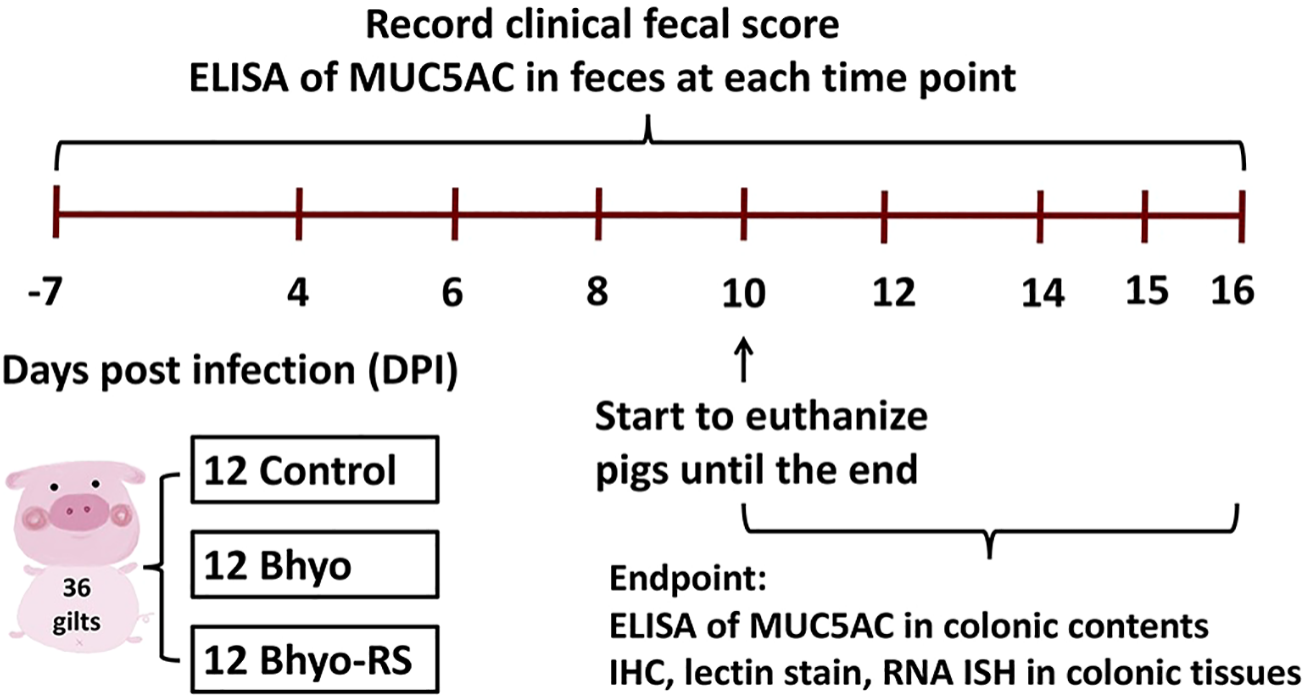
Study design. ELISA to detect MUC5AC detection in feces was performed on samples obtained from DPI-7, and on samples obtained every other day from DPI 4 to the day of euthanasia or at the end of the study on DPI 16. Formalin-fixed, paraffin-embedded tissue samples from the apex of the spiral colon were used for IHC, lectin stain, and RNA-ISH.

The experimental design is shown in **Figure 1**. Fecal samples and paraffin-embedded, formalin-fixed tissue blocks of the spiral colon were obtained from a total of thirty-six gilts from a previous animal inoculation experiment. (Helm et al., 2020) The pigs were from a commercial source with no known history of *Brachyspira*-associated disease, and all of them had been individually confirmed as culture-negative for *B. hyodysenteriae* before their enrollment in the inoculation experiment. The pigs were separated into three groups as previously described (Helm et al., 2020): (1) *B. hyodysenteriae* negative, fed a typical corn and soybean meal diet that contains 20% of DDGS (Control, n=12), (2) *B. hyodysenteriae* challenged, fed a typical corn and soybean meal diet that contains 20% of DDGS (Bhyo, n=12), and (3) *B. hyodysenteriae* challenged, fed a highly fermentable fiber diet including 5% sugar beet pulp and 5% resistant starch diet (Bhyo-RS, n=12). The *B. hyodysenteriae* (B204) strain used was originally recovered from a clinical case of SD in 1972 and was obtained from the culture archive at the Iowa State University Veterinary Diagnostic Laboratory (ISU VDL). On days post-inoculation (DPI) 0 and 1, pigs were inoculated with B. *hyodysenteriae* or sham as previously described (Helm et al., 2020). Fecal samples were collected every other day until euthanized or the end of the study (DPI 16), and were frozen and stored at -80°C until further ELISA experiment. Clinical fecal consistency score was evaluated and scored each morning as in previous experiments (Wilberts et al., 2014b; Helm et al., 2020). Briefly, normal fecal consistency scores 0, soft but formed feces score 1, semisolid feces score 2, and liquid to watery feces score 3. An additional 0.5 point was added when there was presence of mucus and/or blood to a total maximum score of 4. Pigs were euthanized within 72 hours once clinical SD was observed (as indicated by a score of 3.5 or greater), or at the end of the study on DPI 16 (between DPI 10 and 16). Euthanasia and necropsy from all three groups were performed on five different days.

### 2.2 Enzyme-linked immunosorbent assay

ELISA to detect MUC5AC detection in feces was performed on samples obtained from DPI-7, and on samples obtained every other day from DPI 4 to the day of euthanasia or at the end of the study on DPI 16. ELISA to detect MUC5AC was also performed on colon content samples obtained at euthanasia. For each sample, about 60 mg of feces were suspended and mixed in 600μL buffer solution (PBS) and centrifuged at 1000g for 20 minutes. The supernatants were collected, used to determine total protein concentrations, and stored at -80°C until further use. Total protein concentrations were determined with a bicinchoninic acid (BCA) assay (Thermofisher Scientific, Waltham, MA). A commercialized ELISA kit was used in the study (Cat No. MBS028569, MyBioSource, San Diego, CA) and all procedures were performed according to the manufacturer’s manual. All ELISA reagents and frozen samples were brought to room temperature before the experiments. Briefly, the ELISA test was started by adding 50μl of 6 standards and all the sample supernatants individually in duplicates to the 96-well microliter plate that was included in the assay kit, followed by adding 100μL HRP-Conjugate Reagent to each well, incubated for 60 minutes at 37°C and washed with the provided buffer. A blank well with no reagents was set as well. Two chromogen solutions with amounts of 50μL were added to every well and incubated for 15 minutes at 37°C and protected from light. An amount of 50μL stop solution was added to every well, and the optical density (OD) of each test and standards were measured at 450nm using a Cytation 5 Hybrid Multi-Mode Reader (BioTek Instruments Inc., Winooski, VT) within 15 minutes, and data are presented as ng/mg isolated protein.

### 2.3 Gross pathology and histopathology

Gross lesion observation was conducted as previously described with slight modification to include a semi-quantitative assessment of mesocolonic edema and mucosal necrosis with a total maximum gross lesion score of 15 (Wilberts et al., 2014b). Briefly, the intestinal tract was observed and evaluated for the presence of mesocolonic edema (none 0, mild 1, moderate 2, severe 3). The full length of the cecal and colonic lumens was exposed and evaluated for the presence and distribution of mucosal necrosis (none 0, multifocal 1, diffuse 2), luminal mucus (none 0, mild 1, moderate 2, severe 3), mucosal hemorrhage (none 0, mild 1, moderate 2, severe 3), and fibrinous exudate (none 0, mild 1, moderate 2, severe 3). Lesion distribution was recorded (cecum, the base of the spiral colon, the mid-centripetal portion, apex of the spiral colon, and the descending colon; absent 0, present 1). Tissues from the spiral colon were collected and sectioned at 4 μm, stained with hematoxylin and eosin, and examined for microscopic lesions. Sections of the ileum were evaluated for the presence of lesions indicative of *Lawsonia intracellularis* or *Salmonella* spp. infection. Crypt depth and neutrophil count were estimated on the section of the apex of the spiral colon as previously described (Lin et al., 2021). Briefly, mean neutrophil count in the lamina propria on the sections from the apex of the spiral colon were counted and obtained from ten 400x fields. Crypt depth was measured on the same sections by using the ruler function in the commercial software package (CellSens ver. 1.18, Olympus USA). Mean crypt depth was obtained from ten measurements of crypts that were orientated perpendicularly to the mucosa for each case.

### 2.4 Lectin histochemistry

#### 2.4.1 *Lotus tetragonolobus* lectin (LTL) stain

Formalin-fixed, paraffin-embedded tissue samples from the apex of the spiral colon were sectioned at 4 μm onto positively charged slides and baked at 57°C for 2 hours prior to use. Sections were processed by automated staining using the Roche Ventana Medical System DISCOVERY ULTRA with ready-to-use ancillary and detection agents from Ventana (Ventana Medical Systems, Inc., Tucson, AZ). Briefly, sections were deparaffinized at 72°C, incubated with high pH Cell Conditioning 1 (CC1) for antigen retrieval for 24 min at 100°C, and with Inhibitor CM for 12 minutes to inhibit the endogenous peroxidase. Slides were then incubated with biotinylated *Lotus Tetragonolobus* (LTL) lectin at room temperature for 1 hour (dilution 1:100 in phosphate buffer saline (PBS), Vector Laboratories, catalog no. B-1325, Burlingame, CA), which targets α-linked L-fucose. Horse radish peroxidase-streptavidin conjugate (Agilent/Dako, Santa Clara, CA) was applied to slides at room temperature for 40 minutes. Slides were incubated with the DISCOVERY Amp HQ Kit for 12 minutes and then with the anti-HQ HRP Multimer for 12 minutes. Slides were finally visualized using the DISCOVERY ChromoMap DAB Kit (RUO) following the manufacturer’s instructions, counterstained for 8 minutes with HEMATOXYLIN, and mounted.

#### 2.4.2 *Griffonia simplicifolia* lectin (GS-II) stain

Formalin-fixed, paraffin-embedded tissue samples from the apex of the spiral colon were sectioned at 4 μm onto positively charged slides, baked at 57°C for 2 hours, deparaffinized in xylene, and rehydrated in graded alcohol and water baths. Sections were incubated with 3% hydrogen peroxide to inhibit the endogenous peroxidase for 20 minutes, followed by 3 rinses with PBS. Antigen retrieval was attained by microwaving slides (1100-W microwave) in citrate buffer (pH 6.2) for 45 seconds at 100% power to boiling, followed by 5 minutes at 20% power. Slides were then incubated with 1% bovine serum albumin for blocking for 10 minutes at room temperature. After 3 rinses with PBS, slides were incubated with biotinylated *Griffonia simplicifolia* (GS-II) lectin targeting GlcNAc (concentration 25 μg/ml, in PBS containing 0.5 nM CaCl_2_, EY Laboratories, catalog no. BA-2402-2, San Mateo, CA) at room temperature for 1 hour in a humidified chamber. After 3 washes in PBS, slides were incubated with horse radish peroxidase-streptavidin conjugate (Agilent/Dako, Santa Clara, CA) at room temperature for 10 minutes and then rinsed with PBS for 5 minutes. The reaction was visualized using a commercial chromogen (NovaREDTM, Vector laboratories, Burlingame, CA) for 4 minutes at room temperature and rinsed with ultrapure water. Slides were then subjected to a Shandon’s hematoxylin counterstain, placed in Scott’s Tap water for 1 minute, rinsed with ultrapure water, dehydrated through a graded alcohol series, and mounted.

#### 2.4.3 *Sambucus nigra* (SNA) lectin stain

Staining procedures were similar to those previously mentioned for LTL stain. Slides were stained with the biotinylated *Sambucus Nigra* (SNA) lectin at room temperature for 1 hour (dilution 1:100 in VENTANA Antibody Diluent containing casein, Vector Laboratories, catalog no. B-1305-2, Burlingame, CA). The lectin is obtained from elderberry bark, which mainly stains with NeuAc attached to terminal galactose in α-2,6 and to a lesser degree, α-2,3 linkage.

#### 2.4.4 *Maackia Amurensis* II (MAL II) lectin stain

Staining procedures were similar as previously mentioned for LTL stain, but antigen retrieval was attained by using PROTEASE 1 for 4 minutes. Slides were incubated with biotinylated *Maackia Amurensis* (MAL II) lectin at room temperature for 1 hour (dilution 1:100 in PBS, Vector laboratories, catalog no. B-1265-1, Burlingame, CA). The lectin is obtained from their seeds, which stains with NeuAc in an α-2,3 linkage.

### 2.5 Immunohistochemistry

#### 2.5.1 N-glycolylneuraminic acid (NeuGc) stain

Staining was performed using a polyclonal chicken anti-Neu5Gc antibody (BioLegend, catalog no. 146901, San Diego, CA). Staining procedures were similar to GS-II stain but with some modifications. No antigen retrieval was performed in this protocol, and slide blocking of non-specific binding sites was performed by application of the blocking solution (1:40 dilution in pH4 PBS) from the same antibody kit. After 3 rinses with PBS, slides were incubated with the primary anti-Neu5Gc antibody at room temperature for 1 hour in a humidified chamber. The primary antibody for Neu5Gc was dilated to 1: 2000 in the diluted blocking solution. Slides were washed with PBS and incubated with a peroxidase conjugated donkey anti-chicken secondary antibody (Jackson Immuno Research, catalog no. 703-035-155, West Grove, PA) for 1 hour at room temperature. The secondary antibody was diluted to 1:500 in PBS. Following three times of washes with PBS, slides were incubated with chromogen (NovaREDTM, Vector laboratories, Burlingame, CA) for 4 minutes at room temperature, rinsed with ultrapure water, counterstained, dehydrated, and mounted.

#### 2.5.2 MUC5AC and MUC2

Immunohistochemical stains targeting MUC5AC and MUC2 were performed similarly to the LTL stain but with some modifications. For MUC5AC, antigen retrieval was attained by using high pH CC1 at 100 °CC for 48 minutes. A primary monoclonal mouse anti-human antibody for MUC5AC (Invitrogen, Thermo Fisher Scientific, catalog no. MA5-12178, Waltham, MA) was used with a dilution of 1:100 in VENTANA Antibody Diluent containing casein and slides were incubated at 37°C for 32 minutes. Anti-Mouse HQ and Anti-HQ HRP were applied and incubated for 12 minutes, respectively. For MUC2, antigen retrieval was attained by using the low pH Cell Conditioning 2 at 98°C for 24 minutes. A primary polyclonal rabbit anti-human antibody MUC2 (GeneTex, catalog no. GTX100664, Irvine, CA) was used with a dilution of 1:500 in VENTANA Antibody Diluent containing casein. Slides were incubated with the primary antibody at 37°C for 32 minutes, with Anti-Rabbit-HQ for 12 minutes, and with Anti-HQ HRP for another 12 minutes.

### 2.6 RNA *in situ* hybridization

RNA ISH was performed as previously described (Lin et al., 2021) using chromogenic RNA ISH for IL-17 A (gene ID: 449530, catalog no. 490831, Newark, CA) and the RNAscope 2.5 HD Assay- Red reagents obtained from Advanced Cell Diagnostics (ACD RNAscope, catalog no. 322350, Newark, CA). Appropriate controls were present in every run, including the positive control probe-Ss PPIB (ACD RNAscope) and negative control probe-dapB (ACD RNAscope).

### 2.7 Image analysis

Image analysis of slides labeled with immunohistochemistry, histochemical staining, or RNA ISH was performed as previously described (Lin et al., 2021). For NeuGc stain, five representative 100X images of the full thickness of the colonic mucosa were captured for each slide. For MUC5AC, MUC2, and IL-17A, five representative 400X images of the colonic mucosa, regardless of specific location, were captured separately for each slide. For all other stains except for NeuGc and IL-17A, five representative 400X images of the top half and the bottom half of the colonic mucosa were captured separately for each slide. For immunohistochemical and histochemical staining, captured images were analyzed quantitatively using the Area Quantification module v1.0 within the HALO image analysis platform as previously described (v2.0.1145.19, Indica Labs) (Lin et al., 2021). Briefly, representative staining colors of nuclear stain (blue hematoxylin) and positive signal (brown chromogen) were set as the referenced color baseline. Quantification of the total chromogen staining area was analyzed by the module algorithm as a percentage of positive staining of the region of interest. All staining artifacts or regions of white space were manually excluded from the analyzed area. For NeuGc stain, all the lamina propria areas including lamina proprial inflammatory cells were manually excluded by using the software exclusion tool to quantify only the area of epithelium. For RNA ISH image analysis, the ISH module v2.2 within the HALO image analysis platform (v2.0.1145.19, Indica Labs) was used (Lin et al., 2021). Briefly, the representative density of the nuclear stain and positive signal stain color was specified and set manually. Individual cells in the image were circled and separated by adjusting a series of input data, including nuclear size, nuclear segmentation aggressiveness, cell radius from nuclei, etc. Cells are grouped into five bins (0 to 4+) based on the number of dots and signals per cell. Each image is evaluated for the percentage of cells in each bin. The output is an H-score that reflects a cell-by-cell quantitative result for each image by calculating the total percentage of cells in each bin and reflecting the overall target expression on a scale of 0 to 400 (Jolly et al., 2019).

### 2.8 Statistical analysis

Statistical analysis of ELISA, histopathology, histochemistry, IHC, and RNA ISH data was performed using commercial statistical software packages R (version 4.1.1, R Foundation for Statistical Computing, Vienna, Austria) and GraphPad Prism 9 (GraphPad Software, San Diego, CA). General linear models considering three treatments (Control, Bhyo, Bhyo-RS) were used to detect statistical differences in ELISA results for each day or data from the endpoint, neutrophilic inflammation, crypt depth, area quantification of staining, and the H-score of RNA ISH. The Shapiro-Wilk test was used to examine the normal distribution of the residual. Data were analyzed using the one-way analysis of variance (ANOVA) or the Kruskal Wallis test whenever applicable. Results were expressed as mean with a standard deviation of the means for normally distributed data and as median with interquartile ranges for non-normally distributed data. Tukey’s or Dunn’s tests were used for *post hoc* multiple comparisons whenever applicable. Correlation analysis built out of the residuals was performed in all data pairs of stains, RNA ISH, and ELISA, comparing the values from all animals, and using the Pearson correlation test. Results were shown as a correlation coefficient (*R*) with 95% confidence interval (CI). Statistical significance for all data was set as *P* ≤ 0.05.

## 3 Results

### 3.1 Gross pathology and histopathology

Neutrophil count, crypt depth, and gross lesions were significantly different between groups (*P* < 0.05, **Table 1**, Kruskal-Wallis, **Supplemental Materials**). There were significantly increased neutrophils in the lamina propria of the spiral colon at the apex in the groups of Bhyo and Bhyo-RS compared to the controls (both *P* < 0.05, **Supplemental Materials**). Gross lesions were similar to those previously described (Wilberts et al., 2014b), presenting mostly in the base, middle-centripetal, and apex of the spiral colon comprising variable degrees of mucosal necrosis, mucosal thickening, and fibrinohemorrhagic exudate with excessive mucus production. Gross lesion scores were significantly higher in the two inoculated groups compared to the controls, which did not show any gross lesions (both *P* < 0.05). As previously mentioned (Helm et al., 2020), there were three Bhyo-RS pigs (25%) that did not develop any clinical signs at the end of the study and had soft to semisolid feces at necropsy, in comparison to the Bhyo group that all pigs developed clinical SD. Histopathology in the current study showed that only one of the three pigs did not have characteristic lesions of SD and presented normal length of the colonic mucosa and minimal neutrophil count. The gross lesion score of this animal was 0.

**Table 1:** Histopathologic findings in pigs with and without swine dysentery after *Brachyspira hyodysenteriae* infection and fed a diet containing sugar beet pulp and resistant starch diet in one of the inoculated groups.

	Control (*n*=12)	Bhyo (*n*=12)	Bhyo-RS (*n*=12)	*P* value
**Gross lesion score (IQR)**	0^b^ (0)	10^a^ (2.25)	8.5^a^ (3)	<0.0001
Histopathology				
**Neutrophil count (numbers, IQR)**	3.30^b^ (4.95)	78.90^a^ (39.25)	79.15^a^ (59.7)	<0.0001
**Crypt depth (μm, IQR)**	625.48^b^ (90.74)	1033.60^a^ (112.57)	997.92^a^ (176.67)	<0.0005
Lectin stain(area quantification, %)				
Fucose-LTL crypt base (StdD)	1.53^b^ (2.15)	10.28^a^ (7.16)	6.87^ab^ (6.21)	<0.005
**Fucose-LTL crypt top (StdD)**	0.29^a^ (0.51)	15.83^b^ (8.36)	8.38^c^ (7.53)	<0.0001
**GlcNAc-GS-II crypt base (StdD)**	14.60^b^ (4.42)	7.69^a^ (6.26)	6.17^a^ (4.64)	<0.001
**GlcNAc-GS-II crypt top (IQR)**	3.14^b^ (1.95)	5.23^a^ (2.66)	5.08^a^ (4.68)	<0.005
**NeuAc-SNA crypt base (StdD)**	14.25^b^ (10.13)	2.57^a^ (4.08)	4.36^a^ (6.14)	<0.0001
**NeuAc-SNA crypt top (IQR)**	21.05^b^ (9.69)	4.86^a^ (12.44)	9.65^ab^ (12.67)	<0.0005
**NeuAc-MAL II crypt base (IQR)**	22.12^b^ (4.93)	4.15^a^ (6.04)	7.23^a^ (5.00)	<0.0005
**NeuAc-MAL II crypt top (StdD)**	28.45 (6.60)	28.40 (6.95)	26.62 (9.08)	0.799
Immunohistochemistry(area quantification, %)				
**NeuGc (IQR)**	11.97 (9.24)	8.44 (7.17)	9.91 (12.13)	0.069
**MUC5AC total (IQR)**	0.04^b^ (0.06)	5.78^a^ (5.27)	5.56^a^ (3.86)	<0.0001
**MUC5AC-crypt base (IQR)**	0.11^b^ (0.13)	0.63^a^ (0.22)	0.76^a^ (1.16)	<0.0005
**MUC5AC-crypt top (IQR)**	0.11^b^ (0.13)	8.87^a^ (5.40)	8.98^a^ (8.41)	<0.0001
**MUC2 total (StdD)**	30.10 (7.09)	32. 63 (5.73)	33.39 (5.88)	0.412
**MUC2 crypt base (StdD)**	32.35^b^ (6.54)	23.44^a^ (4.72)	22.25^a^ (6.37)	<0.0005
**MUC2 crypt top (StdD)**	23.03^b^ (5.12)	32.71^a^ (3.88)	32.53^a^ (6.83)	<0.0001
RNA *in situ* hybridization				
**IL-17A (H score, IQR)**	0.33^a^ (0.07)	0.43^a^ (0.25)	0.45^a^ (0.35)	<0.05

Results reflect the mean or median for each group as appropriate for their distribution. Data were analyzed using the one-way analysis of variance or the Kruskal Wallis test whenever applicable.

RS, sugar beet pulp and resistant starch diet; Bhyo, *Brachyspira hyodysenteriae*; IQR, Interquartile range; StdD, Standard deviation; NeuGc, N-glycolylneuraminic acid; NeuAc, N-acetylneuraminic acid; SNA, Sambucus nigra; GlcNAc, N-acetylglucosamine; GS-II, Griffonia simplicifolia II; MUC5AC, mucin 5AC; MUC2, mucin 2; IL-17A, interleukin 17A.

^abc^ The groups not marked by the same letters are significantly different in the post hoc multiple comparisons, while groups marked with the same letters are not significantly different.

### 3.2 Lectin histochemistry

#### 3.2.1 *Lotus tetragonolobus* lectin (LTL) stain

**Figure 2:**
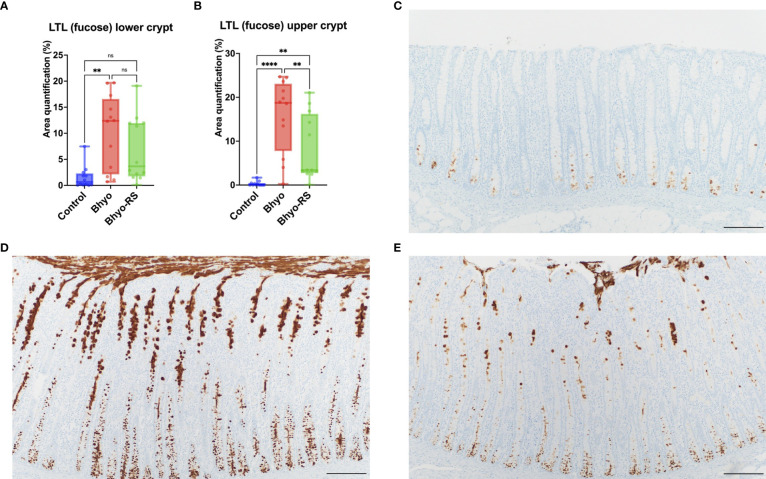
Staining intensity of LTL targeting α-linked L-fucose was significantly different between the three groups **(A, B)** and was low in the control **(C)**. Pigs from the Bhyo group **(D**, *P* < 0.05) had higher intensity of LTL throughout the full thickness of the mucosa compared to the controls **(C)**, and the Bhyo-RS group had a higher staining intensity tendency in the lower mucosa **(A**, *P*= 0.065) and significantly higher staining intensity in the upper colonic mucosa compared to the controls (**B, E** *P* < 0.05). Bar = 200 µm. **, *P* ≤ 0.01; ****, *P* ≤ 0.0001; ns, not significant.

Staining intensity of LTL targeting α-linked L-fucose, in both the upper and lower half of the colonic mucosa, was significantly different between the three groups (**Table 1** and **Figures 2A, B**, *P* < 0.05, ANOVA). For the lower half of the colonic mucosa, only the Bhyo group (**Figure 2D**) had significantly higher intensity of fucose compared to the controls (**Figures 2A, C**, *P* < 0.05, Tukey’s), while Bhyo-RS group (**Figure 2E**) had a tendency for higher intensity of fucose as compared to the controls (**Figures 2A, C** *P*= 0.065). In the upper half of the mucosa, both inoculated groups had greater intensity than the controls (**Figure 2B**, *P* < 0.05), and the group Bhyo-RS group had less intensity than the Bhyo group (**Figures 2B, E**, *P* < 0.05). Bar = 200 µm.

#### 3.2.2 *Griffonia simplicifolia* lectin (GS-II) stain

**Figure 3:**
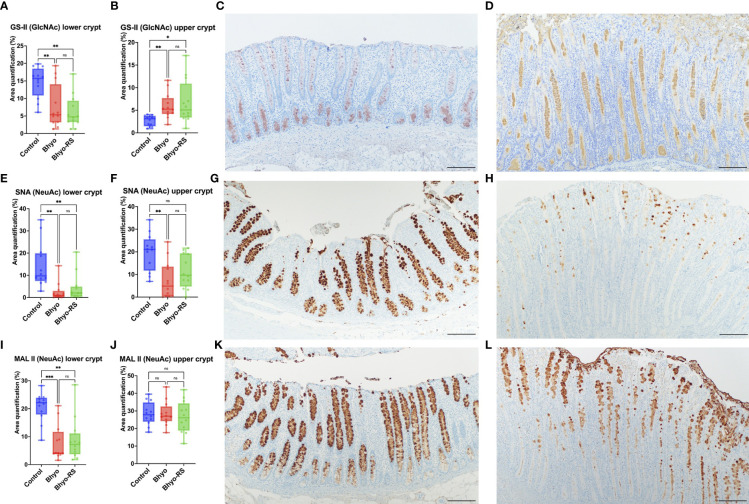
Staining intensity of GS-II targeting GlcNAc was significantly different between the three groups, both in the lower and the upper half of the colonic mucosa (**A, B**, *P* < 0.05). The positivity of GS- II targeting GlcNAc was more confined to the goblet cells in the base of the colonic mucosa in the controls (**C**, *P* < 0.05), while there was abundant GS-II staining within the crypts and in the upper half of the colonic mucosa in the Bhyo and Bhyo-RS **(D)** groups compared to the controls (*P* < 0.05). Staining intensity of SNA targeting NeuAc attached to terminal galactose in an α-2,6 linkage was significantly different between the three groups, both in the lower and the upper half of the colonic mucosa (**E, F**, *P* < 0.05). There was marked positivity in the controls, which was evenly distributed in the goblet cells throughout the full thickness of the mucosa **(G)**. In the Bhyo and Bhyo-RS **(H)** group, there was little to no intensity of α-2,6 linked NeuAc in the lower colonic mucosa **(E)** and little to moderate positivity in the upper half of the mucosa **(F, H)**. Staining intensity of MAL II targeting NeuAc in an α-2,3 linkage was significantly different between the three groups in the lower half (**I**, *P* < 0.05) but not in the upper half of the colonic mucosa **(J)**. Positivity was abundant and well confined to the goblet cells in the controls throughout the full thickness of the colonic mucosa **(K)**, while the Bhyo and Bhyo-RS **(L)** groups had staining intensity mostly in the upper half of the mucosa that was more abundant in the crypt lumens. Bar = 200 µm. *, P ≤ 0.05; **, P ≤ 0.01; ***, P ≤ 0.001; ns, not significant.

Staining intensity of GS-II targeting GlcNAc was significantly different between the three groups both in the lower (*P* < 0.05, ANOVA) and the upper half (*P* < 0.05, Kruskal-Wallis) of the colonic mucosa (**Table 1** and **Figures 3A, B**). The intensity of GS-II was more confined to the goblet cells in the base of the colonic mucosa in the controls (**Figure 3C**), while there was less intensity in the two inoculated groups (**Figure 3A**, *P* < 0.05, Tukey’s). Conversely, the upper half of the colonic mucosa had less intensity of GS-II in the controls, while there was abundant GS-II staining within the crypts in the Bhyo and Bhyo-RS group (**Figure 3D**, *P* < 0.05, Dunn’s).

#### 3.2.3 *Sambucus nigra* lectin (SNA) stain

Staining intensity of SNA targeting NeuAc attached to terminal galactose in an α-2,6 linkage was significantly different between the three groups both in the lower (*P* < 0.05, ANOVA) and the upper half (*P* < 0.05, Kruskal-Wallis) of the colonic mucosa (**Table 1** and **Figures 3E, F**). There was marked staining intensity in the controls, which was evenly distributed in the goblet cells throughout the full thickness of the mucosa (**Figure 3G**), while there was little to no staining in the lower colonic mucosa in the Bhyo and Bhyo-RS group (**Figure 3H**, *P* < 0.05, Tukey’s). In the upper half of the colonic mucosa, the intensity was occasionally detected in the goblet cells and crypts of the Bhyo and Bhyo-RS group (**Figure 3H**). The Bhyo group had significantly lower staining intensity compared to the controls (**Figure 3F**, *P* < 0.05, Dunn’s), while Bhyo-RS had no difference from the controls.

#### 3.2.4 *Maackia Amurensis* II (MAL II) lectin stain

Staining intensity of MAL II targeting NeuAc in an α-2,3 linkage was significantly different between the three groups in the lower half of the colonic mucosa (*P* < 0.05, Kruskal-Wallis, **Table 1**), and both the two inoculated groups had lower staining intensity (**Figure 3I**, *P* < 0.05, Dunn’s). There was no difference in the MAL II expression in the upper half of the mucosa between the three groups (**Figure 3J**). However, the staining was more confined to the goblet cells in the controls (**Figure 3K**) while intensity of MAL II was commonly seen in the crypt lumens in the Bhyo and Bhyo-RS group (**Figure 3L**).

### 3.3 Immunohistochemistry

**Figure 4:**
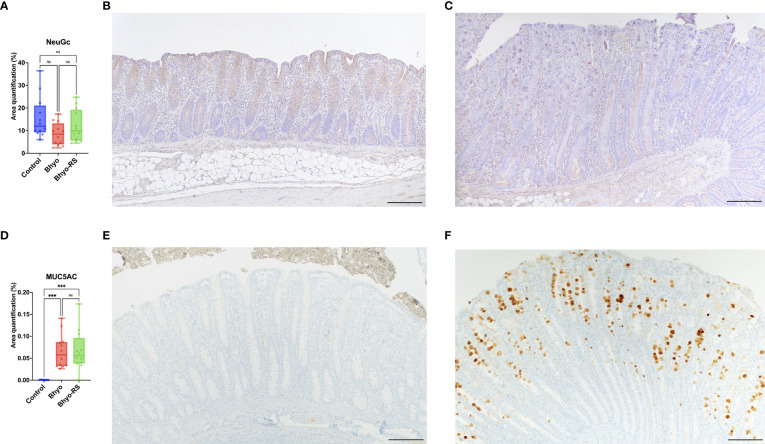
No significant difference in the total expression of NeuGc was detected between the three groups **(A)**. Controls slightly tend to show abundant positive staining within goblet cells and epithelial cytoplasm **(B)**, whereas positivity in pigs infected with *B.hyodysenteriae* was often presented within crypt lumens and less in the cytoplasm **(C)**. MUC5AC expression was significantly different between the three groups (**D**, *P* < 0.05). There was a marked *de novo* expression of MUC5AC in the Bhyo and Bhyo-RS **(F)** groups compared to the controls (**E**, *P* < 0.05). Bar = 200 µm. ***, P ≤ 0.001; ns, not significant.

**Figure 5:**
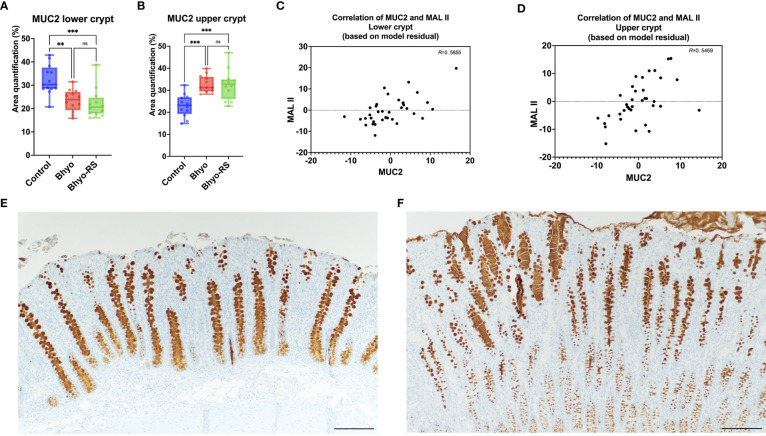
MUC2 expression was significantly different between the three groups, both in the lower and the upper half of the colonic mucosa **(A, B)**, *P* < 0.05). There was a moderate correlation between the expression of MUC2 and MAL II **(C)**, lower half, *R*=0. 5855, *P* < 0.05, and upper half, **(D)**, *R*=0. 5469, *P* < 0.05). There was more MUC2 expression in the lower half of the colonic mucosa in the controls **(E)**, while the Bhyo **(F)** and Bhyo-RS groups had more expression in the upper halves (*P* < 0.05). Bar = 200 µm. **, P ≤ 0.01; ***, P ≤ 0.001; ns, not significant.

No significant difference in total expression of NeuGc was detected between the three treatment groups (**Table 1** and **Figure 4A**, *P*=0.069, Kruskal-Wallis), but subtle differences in staining distribution were observed. The controls showed abundant positive staining within goblet cells and epithelial cytoplasm (**Figure 4B**), whereas the Bhyo (**Figure 4C**) and Bhyo-RS group had similar staining patterns but positivity was often present within crypt lumens and less in the cytoplasm. MUC5AC expression was significantly different between the three groups (*P* < 0.05, Kruskal-Wallis), with Bhyo and Bhyo-RS both higher than the controls (**Figures 4D–F**, *P* < 0.05, Dunn’s) regardless of the location (**Supplemental Figure**). For MUC2 expression, when quantifying the expression of the full thickness of the colonic mucosa, there was no difference between the groups (*P*=0.412, **Supplemental Figure**). When quantifying the expression of MUC2 separately in the lower and upper halves of the colonic mucosa, the controls (**Figure 5E**) had abundant MUC2 expression in the lower half while the Bhyo (**Figure 5F**) and Bhyo-RS group had more expression in the upper halves (**Figures 5A, B**, *P* < 0.05, Tukey’s, **Table 1**). There was a moderate positive correlation between the MUC2 and MAL II both in the lower (*R*=0. 5855, 95% CI = [0.3182, 0.7666], *P* < 0.05) and upper half (*R*=0. 5469, 95% CI = [0.2662, 0. 7421], *P* < 0.05) of the colonic mucosa (**Figures 5C, D**). High or moderate positive correlations were found between MUC2 and GS-II (*R*=0. 6434, 95% CI = [0.3993, 0. 8024], *P* < 0.05), and MUC2 and SNA (*R*=0. 4062, 95% CI = [0.0897, 0. 6483], *P* < 0.05) in the lower halves of the colonic mucosa. There was a moderate positive correlation between neutrophil count and MUC5AC expression (*R*=0. 5169, 95% CI = [0.2269, 0.7227], *P* < 0.05, **Supplemental Figure**).

### 3.4 RNA *in situ* hybridization

**Figure 6:**
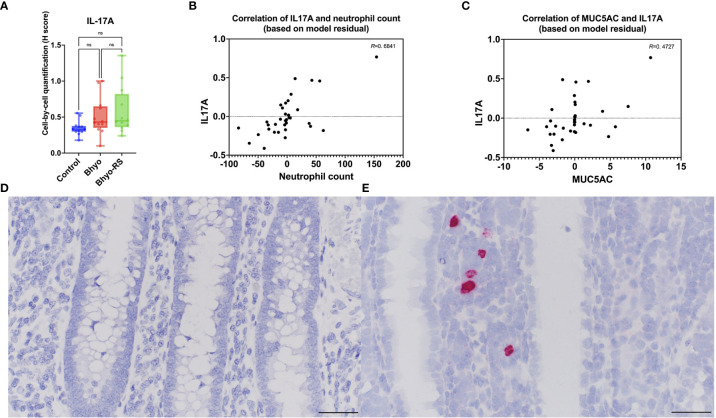
IL-17A expression was significantly different between the three groups (*P* < 0.05). The *post hoc* Dunn’s test showed (**A**, *P*=0.057) showed that the Bhyo-RS group **(E)** had a tendency of higher IL-17A expression compared to the controls **(D)**. A moderate correlation was found between neutrophil count and IL-17A expression (**B**, *R*=0. 6841, *P* < 0.05), and there was a moderate correlation between IL-17A and immunohistochemical MUC5AC expression (**C**, *R*=0. 4727, *P* < 0.05). Bar = 50 µm. ns, not significant.

Colonic IL-17A expression using RNA ISH significantly differed between the three groups (*P* < 0.05, **Table 1**, Kruskal-Wallis), but the *post hoc* Dunn’s test showed that Bhyo-RS group (**Figures 6A, E**) had a tendency of higher IL-17A expression compared to the controls (**Figures 6A, D**, *P*=0.057), and the Bhyo group and the controls did not differ (**Figure 6A**, P=0.151). A moderate correlation was found between neutrophil count and IL-17A expression (*R*=0. 6841, 95% CI = [0.4586, 0.8268], *P* < 0.05, **Figure 6B**), and there was a moderate correlation between IL-17A and immunohistochemical MUC5AC expression (*R*=0. 4727, 95% CI = [0.1707, 0.6935], *P* < 0.05, **Figure 6C**).

### 3.5 Enzyme-linked immunosorbent assay

**Figure 7:**
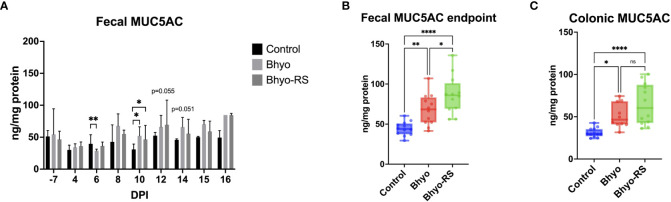
Significant differences in fecal MUC5AC concentration measured by ELISA was found on DPI 6 (**A**, *P* < 0.05, ANOVA) and DPI 10 (**A**, *P* < 0.05, Kruskal-Wallis). On DPI 12 (P=0.055, Kruskal-Wallis) and DPI 14 (*P* = 0.051, Kruskal-Wallis), there was a trend of an overall difference between groups. The two inoculated groups had significantly higher fecal MUC5AC concentrations than the controls on DPI 10 (A, *P* < 0.05, Dunn’s). When comparing the endpoint fecal MUC5AC and colonic MUC5AC concentrations, the two inoculated groups have significantly increased endpoint fecal MUCAC and colonic MUC5AC concentrations (**B, C**, *P* < 0.05, Tukey’s) compared to the controls. Fecal MUC5AC concentrations from the Bhyo-RS group were higher than the Bhyo group (**B**, *P* < 0.05, Tukey’s). There was weak to no correlation found between fecal MUC5AC (ELISA), colonic MUC5AC (ELISA) concentrations, and immunohistochemical MUC5AC expression on the colonic tissues. *, *P* ≤ 0.05; **, *P* ≤ 0.01; ****, *P* ≤ 0.0001; ns, not significant.

Fecal MUC5AC concentrations from DPI-7 to DPI 16 are presented in **Figure 7**. There were significant differences in fecal MUC5AC concentration on DPI 6 (*P* < 0.05, ANOVA) and DPI 10 (*P* < 0.05, Kruskal-Wallis). On DPI 12 (P=0.055, Kruskal-Wallis) and DPI 14 (*P* = 0.051, Kruskal-Wallis), there was a trend of an overall difference between groups. On DPI 6, the control group had higher MUC5AC in the feces than the Bhyo group (*P* < 0.05, Tukey’s). On DPI 10, the two inoculated groups had significantly higher fecal MUC5AC concentrations than the controls (*P* < 0.05, Dunn’s). The pairwise comparison test did not show a difference between group pairs on DPI 14. Correlation tests revealed a strong positive correlation between the clinical fecal score on DPI 8 and fecal MUC5AC but a weak to a very weak correlation between them on other days. Colonic MUC5AC concentration was moderately positively correlated to the clinical fecal score on the day when the pigs were euthanized (*R*=0.4733, 95% CI = (0.1608, 0.6995], *P* < 0.05). Given that pigs were euthanized on five different days and had different endpoints, fecal MUC5AC concentrations of each pig at the study endpoint were selected and compared with colonic MUC5AC concentrations (ELISA) and MUC5AC expression in colonic tissue samples (IHC). The two inoculated groups have significantly increased endpoint fecal MUCAC and colonic MUC5AC concentrations (**Figures 7B, C**, *P* < 0.05, Tukey’s) compared to the controls, and fecal MUC5AC concentrations from the Bhyo-RS group were higher than the Bhyo group (**Figure 7B**, *P* < 0.05, Tukey’s). There were weak to no correlations found between fecal MUC5AC (ELISA), colonic MUC5AC (ELISA) concentrations, and immunohistochemical MUC5AC expression on the colonic tissues.

## 4 Discussion

Utilizing samples from a *B.hyodysenteriae* infection study previously described (Helm et al., 2020), the aims of this study were to describe the alteration of colonic glycosylation and IL-17A expression during acute SD, to determine the diagnostic value of using fecal MUC5AC ELISA to predict SD development, and to determine if highly fermentable dietary fiber has any impact on any of these changes. In the previous study, although not protective, replacing 10% DDGS (insoluble fiber) with highly fermentable 5% sugar beet pulp and 5% resistant starch delayed the onset of clinical SD and reductions of average daily gain.

Protein glycosylation is an essential posttranslational modification by adding glycans (sugars) to specific amino acid sites of proteins (Moran et al., 2011). Lectins are carbohydrate-binding proteins that resemble an antibody and can bind to glycan units depending on their specific affinity. Our results showed that lectin LTL targeting fucose was barely stained in the control pigs, while there was moderately increased staining intensity in the inoculated pigs throughout the colonic mucosa. Fucose is an important nutrient for the colonization of microbes and the established microbiota, which protect the gastrointestinal mucosa (Garber et al., 2021). However, fucose can also be utilized by pathogens such as *Salmonella* Typhimurium (Ng et al., 2013; Suwandi et al., 2019) and *Campylobacter jejuni* (Garber et al., 2020), or serve as a receptor for adhesins of bacteria like *Helicobacter pylori* (Boren et al., 1993; Ilver et al., 1998). Previous studies using tandem mass spectrometry have found decreased fucosylation in the recovered mucin from pigs infected with *B. hyodysenteriae*, and an abundance of fucosylated structures in mucin from healthy pigs suggesting fucose may not be heavily involved in disease pathogenesis (Venkatakrishnan et al., 2017). However, fucose has also been shown to have a chemotactic effect on *B. hyodysenteriae* (Kennedy and Yancey, 1996; Quintana-Hayashi et al., 2021). Herein, we demonstrated an overall increased fucosylation in the colonic tissues of pigs with SD. It may represent a local increased chemotactic activity for *B. hyodysenteriae* with possible positive feedback reactions, or it may represent an increased need for fucose utilization by either *B. hyodysenteriae* or other commensal bacteria. The colonic mucus layer comprises the inner and outer layers (Johansson et al., 2011). The inner layer is secreted by goblet cells, devoid of bacteria, closely attached to the epithelium, and converts to the outer layer. The thickness of the outer layer is twice as thick as the inner layer and provides a microhabitat of the commensal flora (Johansson et al., 2011). The differing results of fucosylation among studies may be related to different detection methods or different disease stages. In previous studies, they recovered and purified colonic mucins from five pigs by scraping the mucosal surfaces of thawed frozen colonic tissues at day 40 post-inoculation (Quintana-Hayashi et al., 2015). The number of days from the start of clinical SD to autopsy varied from 1 to 28 days. Recovered mucins were analyzed using a porous graphitized carbon liquid chromatography tandem mass spectrometry (Quintana-Hayashi et al., 2015; Venkatakrishnan et al., 2017). In contrast to the previous studies, we used lectin stain to directly localize fucose in colonic tissues obtained from pigs with acute SD, which was detected within crypts and goblet cells. Mucin production and degradation are dynamic and associated with the microbiota (Hoskins and Boulding, 1981). Recovered mucin subjected to mass spectrometry and lectin staining targeting mucin in colonic tissues in our study likely reflects different stages and statuses of the mucin microenvironment. Mucin is formed in the crypt goblet cells during migration from the crypt bottom to the crypt opening and luminal surface (Johansson et al., 2011). Once the mucin reaches the lumen, mucin may be degraded by bacterial enzymes or utilized. The commensal microflora and host likely use glycan attachments for mutual selection of each other to maintain an optimal symbiotic relation (Gagneux and Varki, 1999; Johansson et al., 2011). The pigs in our study were euthanized within 72 hours once clinical SD was observed to ensure that most of the pigs were within the acute stage of SD. As a result, the different time points of sample collection may greatly affect the mucin composition, and the mucin in the colonic tissues may not reflect the mucin composition that was analyzed by tandem mass spectrometry from previous studies. It is noteworthy that *Bacteroides thetaiotaomicron*, *B. fragilis*, and *B. vulgatus* all possess fucosidase. The former cleaves fucose from mucin and enables the gastrointestinal pathogen enterohaemorrhagic *Escherichia coli* to sense fucose and modulate its virulence (Pacheco et al., 2012). *B. fragilis* has been shown to facilitate the growth and invasion of *Campylobacter jejuni* in the presence of mucins (Luijkx et al., 2020), and *B. vulgatus* potentially helps *C. jejuni* metabolizes sugars (Garber et al., 2020). Since *B. vulgatus* has been shown to potentially serve as a co-pathogen in the SD development (Harris et al., 1978; Whipp et al., 1979), and the growth of *B. hyodysenteriae* is not specifically enhanced in the presence of fucose (Quintana-Hayashi et al., 2019), the role of increased local fucose expression in SD and its interaction with other commensals warrants further investigation.

The lectin GS-II targeting terminal GlcNAc showed spatial distribution differences with more positivity in the lower half of the colonic crypts in the controls, while pigs with SD had a moderate staining intensity of GlcNAc throughout the mucosa but often accumulated it in the crypt lumens of the upper half of the mucosa. The turnover of the intestinal mucus layer is a dynamic and delicate process. In the colon, crypt stem cells divide in the crypt bottom, differentiate into goblets cells, and migrate towards the openings of crypts (Johansson et al., 2011). Superficial goblet cells at the crypt openings secrete mucin continuously to form the inner mucus layer, while goblet cells in the upper part of the colonic crypts secrete mucin in response to stimuli (Specian and Neutra, 1980; Pelaseyed et al., 2014). It is possible that in the presence of *B. hyodysenteriae*, a continuous GlcNAc secretion into the lumen is stimulated and serves as a nutrient source (Quintana-Hayashi et al., 2019). While the secreted mucus can be seen within the upper half of the mucosa, there is likely temporary exhaustion of goblet cells in the lower half of the mucosa that results in inadequate replenishment of new goblet cells.

The two lectins targeting NeuAc in α-2,6 linkage and α-2,3 linkages (SNA and MAL II) showed marked staining intensity, which was evenly distributed throughout the full thickness of colonic mucosa in the control pigs, only sparing the very basal crypt. NeuGc was moderately expressed in the goblet cells and cytoplasm of the colonic epithelium in healthy control pigs. Conversely, the Bhyo group had marked depletion of NeuAc in α-2,6 linkage (SNA) both in the lower and upper halves of the colonic mucosa. NeuAc in α-2,3 linkage (MAL II) was also depleted in the lower half in pigs inoculated with *B. hyodysenteriae* compared to the controls but was moderately stained and accumulated within the crypt lumens in the upper half of the colonic mucosa. For NeuGc, there was a tendency of decreased expression in the two infected groups with more staining within the crypts and less in the cytoplasm. Our results of an overall decrease or no differential staining intensity of sialic acids in pigs infected with *B. hyodysenteriae* were surprising and different from what we expected based upon available literature as glycan chains in pigs infected with *B. hyodysenteriae* were previously shown to contain more NeuGc, a proposed binding site for *B. hyodysenteriae*, and growth of *B. hyodysenteriae* is enhanced in the presence of free sialic acid and GlcNAc (Venkatakrishnan et al., 2017; Quintana-Hayashi et al., 2019). However, it is possible that local colonic depletion of sialic acids in pigs with SD was due to excessive excretion and overall increased consumption of sialic acids by either *B. hyodysenteriae* or other bacteria in the colonic environment. Similar to fucose, the mucin being produced and demonstrated here in the colonic tissues may not completely correspond to the composition of mucin that was analyzed by tandem mass spectrometry previously. Furthermore, there was some mucin being expelled into the lumen of the intestine and covering the epithelium, which we did not quantify. Quantification of the luminal mucin seemed relatively impractical and may differ artifactually unless a very standard tissue collection is performed to preserve the mucus *in situ*. The potential for washout during histologic processing makes the comparison of luminal mucin expression more difficult. Sialic acids are excellent sources of carbon and energy for intestinal commensals and pathogens. As *B. hyodysenteriae* does not harbor sialidase (Quintana-Hayashi et al., 2019), other bacterial species such as *Prevotella* spp., *Bacteroides* spp., *Fusobacterium* spp., or *Ruminococcus* spp (Haines-Menges et al., 2015) that have sialic acid catabolism genes may contribute to sialic acid cleavage, which have been observed to be enriched in pigs with SD (Burrough et al., 2015; Helm et al., 2021). We have characterized increased sialomucin in the colonic mucosa of pigs infected with *B. hyodysenteriae* in our previous studies (Lin et al., 2021), and the results of the current study suggest this increase in sialomucin was not related to any of the specific glycans examined. Differences between these studies may also be a result of dietary differences, and different image quantification methods as herein we spatially quantified the mucin expression in the upper and lower halves of the mucosa.

Secretory mucin MUC5AC is a heavily glycosylated high molecular weight protein that is normally present in the porcine respiratory tract (Kim et al., 2011), stomach (Quintana-Hayashi et al., 2015), and gall bladder (Ushio et al., 2020), but not in the colon of healthy pigs (Wilberts et al., 2014b). Previous studies have shown *de novo* MUC5AC expression in the colon of pigs infected with *B. hyodysenteriae* (Wilberts et al., 2014b; Quintana-Hayashi et al., 2015; Lin et al., 2021). While the present diagnostic tools for SD mostly help identification of the causative *Brachyspira* spp. once pigs become clinical, markedly increased MUC5AC expression in pigs with SD might be a potential diagnostic tool that could help predict or precede disease development, or monitor disease progression. In our study, there was a numerical variation of fecal MUC5AC in the controls on different days. Clinical fecal scores were only correlated with fecal MUC5AC concentrations on DPI 8. Although colonic MUC5AC concentrations were moderately correlated with clinical fecal scores, there was no correlation between fecal, colonic MUC5AC (ELISA) concentrations and IHC for MUC5AC expression in the colonic tissues. All these results suggest that fecal MUC5AC may not directly reflect the clinical status of SD, which may be due to the presence of MUC5AC from extra-intestinal sources in the feces such as gallbladder, stomach, or respiratory tract (if being coughed up and swallowed). The lack of correlation between colonic and fecal MUC5AC ELISA also implies that MUC5AC in feces or colonic content is likely, not homogenized, further indicating the lack of value of MUC5AC as a diagnostic tool. However, when examining endpoint fecal MUC5AC and colonic MUC5AC concentrations, as well as results from DPI 10, the two inoculated groups had significantly higher MUC5AC production. This confirmed that during overt clinical disease progression of SD, fecal MUC5AC was still in line with previous studies, and pigs with SD have higher fecal MUC5AC concentrations. It is noteworthy that on DPI 6, the control group had a higher fecal MUC5AC compared to the Bhyo group. Whether this was an incidental finding or an indication of a transient decrease of baseline MUC5AC excretion from extraintestinal sources early in SD needs further investigation. Our result showed that the temporal concentrations of MUC5AC in feces after inoculation with *B. hyodysenteriae* were variable and were neither predictive of disease onset nor correlated with disease severity. This assay may have some diagnostic value during overt clinical disease; however, the fecal ELISA for MUC5AC used in this experiment is not a reliable monitoring tool for disease progression in SD.

Besides *de novo* expression of MUC5AC in pigs infected with *B. hyodysenteriae*, it has been shown that there is a substantial increase of MUC2 at both the mRNA and protein level (Quintana-Hayashi et al., 2015). We have previously used an immunohistochemical stain targeting MUC2 and found no significant difference in expression between groups (Lin et al., 2021), although the location of staining was different and was mostly present in crypt lumens in pigs infected with *B. hyodysenteriae*. In our present study, we have modified our quantification method and found greater MUC2 expression in the upper half and depletion in the lower half of the colonic mucosa in pigs infected with *B. hyodysenteriae* compared to the control pigs. This distribution pattern is similar to GS-II targeting GlcNAc, which exhibited mucin accumulation in the upper crypt lumens with depletion and inadequate mucin replenishment in the lower part of the mucosa. Such difference could not be detected when quantifying the full thickness and highlighted the need for optimization of the quantification method. MUC2 is the major structural component of the outer mucus layer in the intestine, which build a mucus barrier that separates bacteria from the colon epithelia (Johansson et al., 2008) and has been shown to be associated with antimicrobial activity (Cobo et al., 2015). Fluorescence microscopy has shown that pigs infected with *B. hyodysenteriae* have increased MUC2, but they appeared to be disorganized with a loss of striations parallel to the mucosa (Quintana-Hayashi et al., 2015). Here we found that expression of MUC2 is moderately correlated with one of the sialic acid stains MAL II, suggesting MUC2 potentially harbors abundant NeuAc in α-2,3 linkages. Glycosylation of MUC2 has been shown to be affected by microbiota and regulated by differential expression of glycosyltransferases (Arike et al., 2017). It is likely that the host-pathogen interaction in pigs infected with *B. hyodysenteriae* results in increased MUC2 expression associated with the presence of NeuAc in α-2,3 linkages.

The molecular mechanisms underlying excessive MUC5AC production in pigs with SD are not fully understood but have been associated with neutrophil elastase and IL-17A through *SPDEF, FOXA3*, and mitogen-activated protein kinase 3/extracellular signal-regulated kinase 1 signaling pathway (Quintana-Hayashi et al., 2017). They also demonstrated upregulation of the *IL-17A* gene associated with *de novo* expression of MUC5AC and neutrophil infiltration in colonic tissue in four pigs (Quintana-Hayashi et al., 2017). Herein we showed a tendency of increased IL-17A expression in pigs infected with *B. hyodysenteriae* using RNA ISH, and there were moderate positive correlations between the expression of IL-17A and MUC5AC, and neutrophil count. Such finding further strengthens the role of IL-17A in the mucin production mechanism in pigs infected with *B. hyodysenteriae*. IL-17A is a multifaceted cytokine involved with barrier immunity and it recruits neutrophils through chemokine induction and granulocyte-colony stimulating factor (G-CSF), which acts synergistically with other proinflammatory cytokines such as IL-1, IL-6, and TNF-α. (Mantovani et al., 2011; Ge et al., 2020). The cytokine is mainly produced by CD4 T cells as well as by other cells such as γδ T cells, lymphoid tissue inducible cells, innate lymphoid cells type 3 (ILC3s), and natural killer cells. In our specimens, expression of IL-17A is mostly found in cells with a round contour with round nuclei, which we presume to be lymphocytes, and admixed with aggregates of inflammatory cells in the lamina propria. While pigs with SD usually manifest with mucohemorrhagic, fibrinonecrotic typhlocolitis with aggregates of abundant degenerate neutrophils in the colonic mucosa, such neutrophil recruitment is likely associated with IL-17A. Overall, we confirmed that IL-17A may play a role in the immunoregulatory function in pigs with SD and may affect MUC5AC production.

Diet composition greatly alters disease susceptibility and resolution, which displays an intricate mechanism through alteration of gut microbiome, host-pathogen interaction, and intestinal mucosal barrier (Desai et al., 2016; Makki et al., 2018). It is believed that a highly fermentable fiber diet has a protective role and reduces the incidence and severity of SD in pigs (Helm et al., 2021). We have previously shown that the replacement of insoluble fiber with more highly fermentable fiber sources, i.e., the addition of resistant starch and sugar beet pulp, delayed the disease onset and reductions in average daily gain and apparent total tract digestibility in this group of pigs (Helm et al., 2020). Herein we further investigated the histopathological lesion and local expression of different staining in the colonic tissues to see if there was any difference among the groups. Although testing culture positive for *B. hyodysenteriae*, there were three pigs in the Bhyo-RS treatment group that did not show clinically detectable diarrhea before the end of the study. Among these three pigs, histopathology revealed that one of them did not show any characteristic lesions of SD and had a completely normal mucosa, while the other two did. The major difference in the parameters we measured between the two inoculated groups was that there was less fucosylation in the upper half of the colonic mucosa in the pigs fed a highly fermentable fiber diet. The α1-2 fucosyltransferase (*FUT2*) gene encodes the enzyme that is responsible for adding an L-fucose residue to gut mucus glycans (Bry et al., 1996). About 20% of people lack this gene and lack terminal fucose residues in their distal gut mucin (Kelly et al., 1995), which decreases their susceptibility to *H. pylori* (Magalhaes et al., 2009; Magalhaes et al., 2015) or human noroviruses (Lindesmith et al., 2003). It has been shown that loss of fucosylation on MUC5AC impaired the binding of *H. pylori* BabA adhesin (Magalhães et al., 2016). Despite the lack of correlation between MUC5AC and fucose in our study, it is plausible that fucose and MUC5AC may have some relationship and needs further investigation. It has been shown that mice with and without *FUT2* gene had different alpha diversity and community composition, but a glucose-rich plant polysaccharide-deficient diet exerted a strong effect on the microbiota membership and eliminated the effect of genotype, suggesting a diet-dependent manner of gut microbial alteration (Kashyap et al., 2013). In our study, we observed a decreased fucosylation in the Bhyo-RS group. Without another experimental group composed of pigs only fed the highly fermentable fiber diet, the role of fucose and its association with diet in the development of SD cannot be fully determined. It is possible that decreased fucose is mainly due to feeding a diet that directly lessens fucosylation and changes the gut microbiota environment, which leads to more utilization of fucose by protective symbionts. On the other hand, it is also possible that decreased fucosylation observed in Bhyo-RS treatment group is simply a result of a decreased disease presentation. Since there was no significant association between fucose expression and the neutrophil count or crypt depth, we cannot conclude if the disease mitigation by diet is associated with fucose in the present study. Further exploration of the changes and interactions between diet, mucin fucosylation, *B. hyodysenteriae*, and other colonic commensals is warranted.

The limitation of the present study is that we used formalin-fixed colonic tissues and limited lectin stains to demonstrate different glycosylation patterns, and we lacked a fourth experimental group composed of pigs fed only the highly fermentable fiber diet to determine the major effect. Characterization of fucose using different lectins such as *Ulex europaeus* agglutinin-1 and *Aleuria aurantia* Lectin, or lectins targeting other glycans such as galactose and GalNAc, can be performed. Other fixatives such as Carnoys’s solution, methacarn, and buffered paraformaldehyde or cryopreserved tissue samples can also be tested for better mucin preservation (Röhe et al., 2018).

In conclusion, colonic glycosylation during SD showed an overall increased α-linked L-fucose, overall decreased positivity of α-2, 3 and α-2, 6 linked sialic acids, and spatially different distribution of GlcNAc with more intensity in the upper colonic mucosa within the crypt lumens. Some of these findings are different from previous studies, which may be due to differences between quantification measurement methods, but also highlight the intricate alteration of mucin during acute SD. While a highly fermentable diet has been shown to mitigate the disease and delay the onset of SD, here we demonstrated that such a diet also potentially lessens the fucosylation in pigs infected with *B. hyodysenteriae*. The role of fucose in SD pathogenesis warrants further investigation. Expression of IL-17A is moderately associated with neutrophil count and expression of MUC5AC, strengthening its immunoregulatory function and association with mucin production in pigs with SD. Lastly, fecal ELISA for MUC5AC in our experiment did not show much diagnostic value for disease monitoring in SD but high levels of detection may provide supportive evidence of colitis during overt clinical disease.

## Data availability statement

The original contributions presented in the study are included in the article/**Supplementary Material**. Further inquiries can be directed to the corresponding author.

## Ethics statement

The animal study was reviewed and approved by Iowa State University Institutional Animal Care and Use Committee IACUC protocol #19-170.

## Author contributions

Performed live animal experiments, EH and SL. Performed laboratory experiments, SL and EH. Wrote the manuscript, SL. All authors contributed to the study design, results interpretation, and assisted with manuscript editing.
