# Supplementary figures and images for: Acute infection with Brachyspira hyodysenteriae affects mucin expression, glycosylation, and fecal MUC5AC
Source: Front Cell Infect Microbiol. 2023 Jan 6;12:1042815. doi: 10.3389/fcimb.2022.1042815 (PMC9852840; doi:10.3389/fcimb.2022.1042815)

## MUC2

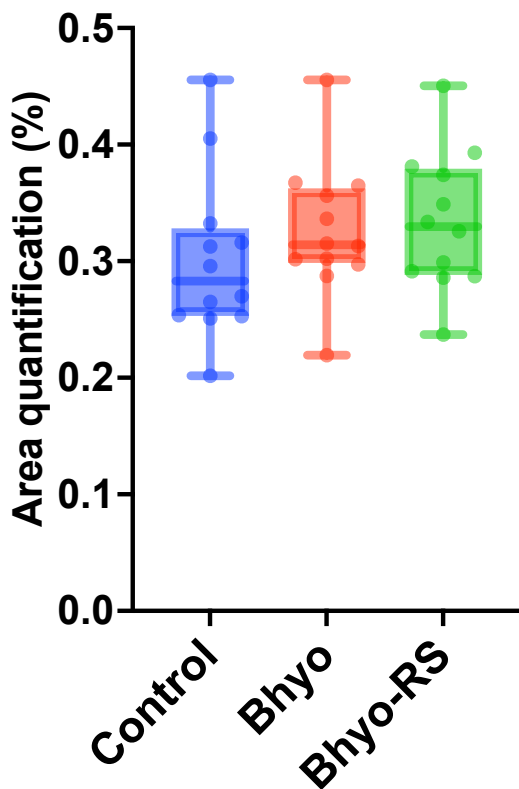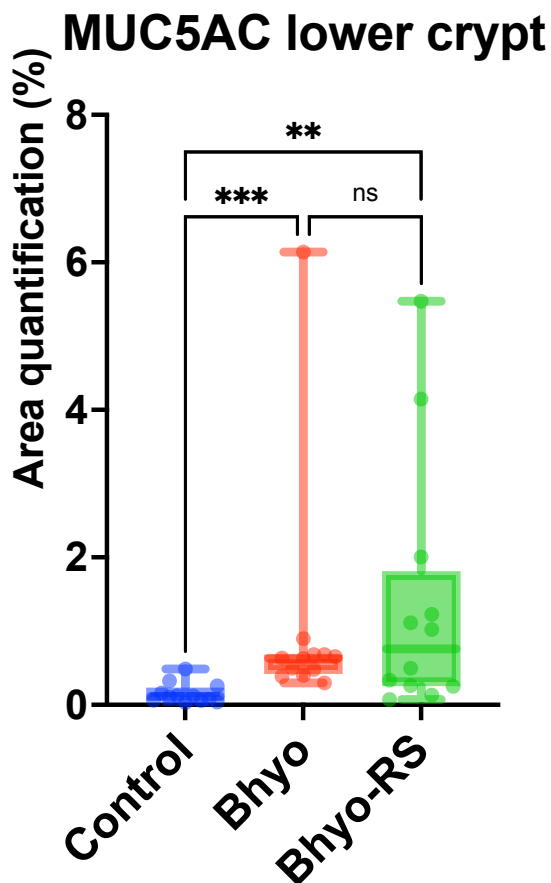

## MUC5AC upper crypt

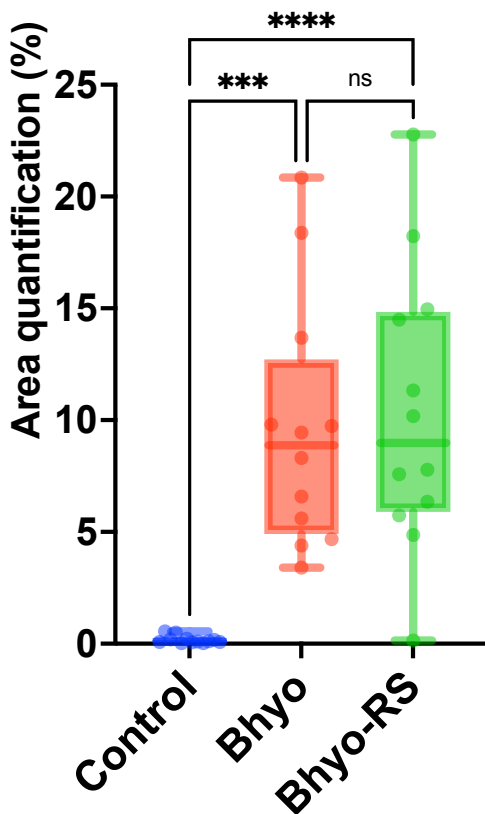

Correlation of MUC5AC and neutrophil count  
(based on model residual)

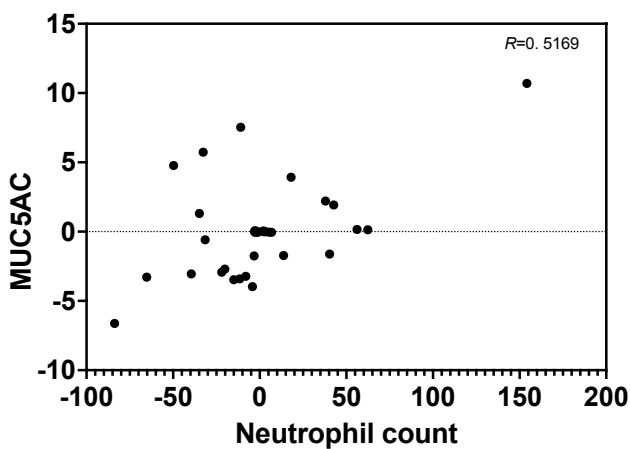

Supplement: Supplementary file 1 [file DataSheet_1.pdf]
